# Traumatic diaphragmatic hernia: delayed presentation with tension viscerothorax – lessons to learn

**DOI:** 10.1308/003588413X13511609955337

**Published:** 2013-03

**Authors:** MS Al Skaini, A Sardar, H Haroon, SM Al Ghamdi, Abdulla Homran, M Ezzedien Rabie

**Affiliations:** Armed Forces Hospitals Southern Region,Saudi Arabia

**Keywords:** Trauma, Hernia, Diaphragm, Tension viscerothorax

## Abstract

Diaphragmatic rupture is a serious complication of thoracoabdominal trauma. The condition may be missed initially. We describe the clinical course of a patient who sustained blunt abdominal trauma in a car accident. His diaphragmatic injury passed unnoticed, to present two years later with left tension viscerothorax, a rarely reported and hardly recognised entity. Nasogastric tube insertion aborted the emergency situation and the hernia was repaired successfully in a semielective setting.

Traumatic diaphragmatic rupture may follow blunt or penetrating thoracoabdominal injury. The resulting hernia may be initially small and easily missed, to enlarge later as more viscera are sucked into the thorax. Obstruction, strangulation and bowel perforation may then follow.^1^ One of the most severe complications of diaphragmatic rupture is tension viscerothorax or gastrothorax.^2^

## Case history

A 30-year-old man, a known asthmatic on irregular medication, presented with chest tightness, shortness of breath, epigastric pain and excessive retching. Two years earlier he had been involved in a car accident. Radiological evaluation at that time, including computed tomography (CT), was reported as normal.

**Figure 1:**
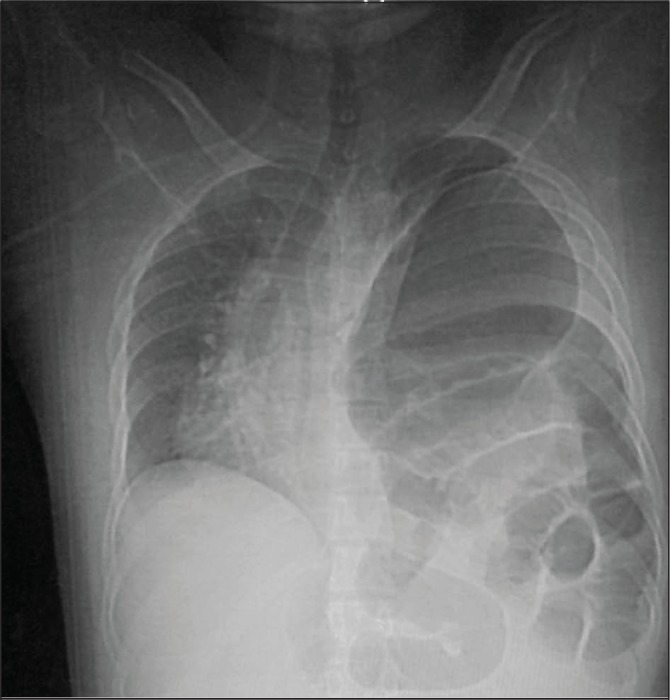
Collapsed left lung with herniation of the stomach and bowel in the left hemithorax with marked mediastinal shift to the right

On present examination, the patient was unable to lie flat, and looked anxious and distressed. His pulse was 125 beats/min, blood pressure 110/70mmHg, respiration rate 25 breaths/min and temperature 37.5°C. His oxygen saturation was 85% on room air and there was a tinge of cyanosis. Abdominal examination showed mild epigastric tenderness, and chest examination showed right tracheal deviation and decreased chest movement with audible bowel sounds in the left side. His blood picture and biochemistry results were within the normal range. A chest x-ray showed a collapsed left lung, and herniation of the stomach and bowel in the left chest, with a marked mediastinal shift, a picture of tension viscerothorax (Fig 1).

**Figure 2:**
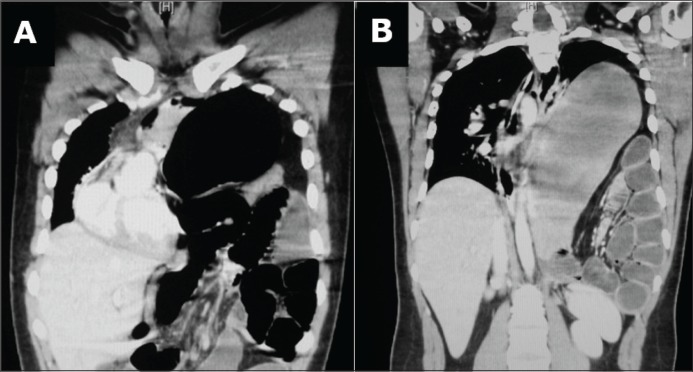
Tension viscerothorax with deviation of the mediastinum to the right side

**Figure 3:**
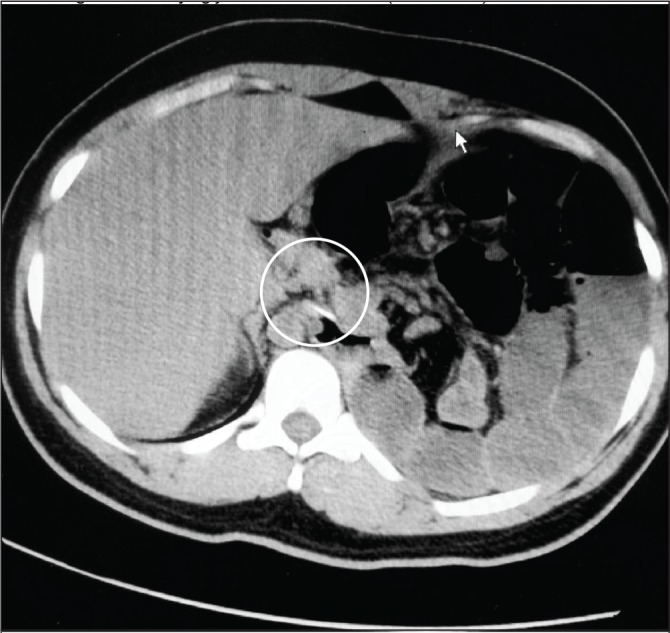
The nasogastric tube lying just below the cardia (white circle)

A nasogastric tube was inserted with difficulty, and this was followed by the passage of air and little bilious fluid. As a result, the distress was relieved and the vital signs almost normalised. Using a nasal cannula, supplemental oxygen was provided and oxygen saturation was maintained until the time of surgery. CT confirmed the diagnosis (Fig 2) and showed the nasogastric tube lying just below the oesophagogastric junction (Fig 3).

**Figure 4:**
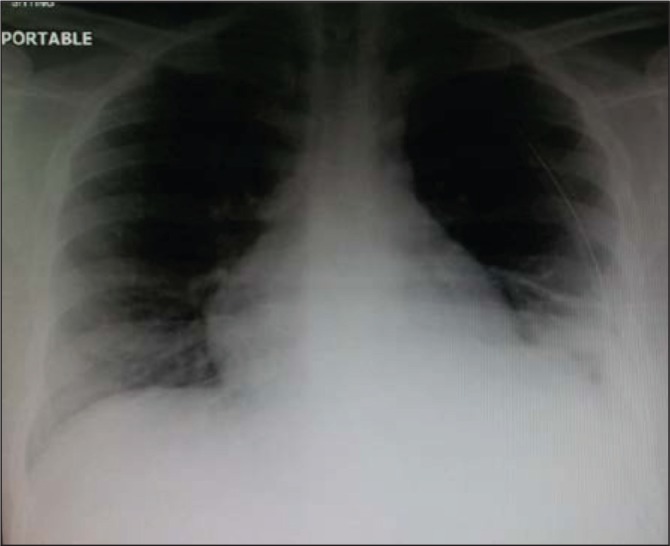
Postoperative chest x-ray showing expansion of the previously collapsed left lung

Owing to waiting for an operation room slot and because the condition was stabilised, surgery was performed in the second day following admission. Repair was carried out via a left anterolateral thoracotomy, through the seventh intercostal space. The herniated viscera were reduced into the abdomen and the diaphragmatic tear was repaired with interrupted polypropylene sutures reinforced with polypropylene mesh. The postoperative recovery was smooth and chest x-ray was satisfactory (Fig 4). The patient was discharged home in good condition and remained so at his outpatient visit.

## Discussion

Victims of road traffic accidents may sustain diaphragmatic rupture when there is a sudden increase in the intra-abdominal pressure, caused by the impact. The injury is seen more frequently on the left side.^1^

Owing to the continuous motion of the diaphragm, which hinders healing, and aided by the negative intrathoracic pressure, the tear enlarges and more abdominal viscera protrude into the thorax, where they may get obstructed or strangulated.^1^ This explains why the injury initially passed unnoticed in our patient, who presented later with tension viscerothorax. Although rare, this trail of events has been reported previously.^3–5^ It may therefore be advisable to obtain a follow-up contrast gastrointestinal series or, preferably, CT scan a few months later, for patients who have sustained thoracoabdominal trauma when no diaphragmatic injury is found initially. Obviously, in patients with severe torso trauma, there should be careful scrutiny to exclude a concomitant diaphragmatic rupture. To this end, helical CT has been claimed to attain high sensitivity and specificity.^1^

When doubt exists and in the absence of a frank indication for laparotomy, thoracoscopy or laparoscopy may be used to visualise occult diaphragmatic injury, with possible endoscopic repair or conversion to open surgery.^6,7^ With laparoscopy, tension pneumothorax may develop owing to a gas leak through the diaphragmatic rent.^8^

In its climax, a diaphragmatic hernia may present with tension viscerothorax, a grave condition that may end with cardiac arrest or bowel gangrene.^9,10^ The clinical and radiological similarity of this condition to tension pneumothorax may create a diagnostic dilemma.^11^ An imprudently inserted chest drain (rather than a nasogastric or orogastric tube) to deflate the intrathoracic stomach^2,9^) will certainly add more complications, as spillage of the visceral contents into the thorax would be inevitable. In the case presented here, the presence of bowel sounds in the chest facilitated the diagnosis before x-ray confirmation.

Immediate decompression of tension pneumothorax, without radiological verification to avoid loss of valuable time, has been a teaching principle. In such conditions, the history of remote thoracoabdominal trauma should direct the attention to the possibility of a tension viscerothorax. In the presence of reasonable doubt and if time permits, a chest x-ray will allow distinction between the two conditions. Although nasogastric or orogastric tube insertion, when successful, decompresses the dilated stomach and restores oxygen saturation,^2^ its insertion may be challenging, with repeated attempts causing further deterioration or cardiac arrest at times.^9^ This difficulty is caused by kinking of the stomach at the diaphragmatic defect. For this reason, the most experienced person in the treating team should attempt its insertion. Additionally, an experienced endoscopist, if available, may attempt tube insertion using a gastroscope. However, as a last resort, percutaneous needle insertion into the stomach may decompress it without spillage.^12^

Our patient presented with many of the features of tension pneumothorax. The presence of audible bowel sounds in the thorax, in addition to the history of old trauma, enabled the correct diagnosis to be reached, which was confirmed with a chest x-ray. A nasogastric tube was inserted just past the oesophagogastric junction and resulted in partial deflation of the stomach. With the aid of supplemental oxygen through a nasal cannula, saturation was maintained. The condition was thus stabilised and the patient was kept comfortable until the time of surgery.

## Conclusions

Despite advances in diagnostic radiology, traumatic diaphragmatic hernia continues to defy early detection in a subset of patients. To avoid this, a high index of suspicion should be maintained while evaluating trauma victims. Follow-up radiology a few months after the injury may recognise those who escaped early detection and, consequently, facilitates timely repair. Tension viscerothorax, which bears many of the features of tension pneumothorax, is a complication of delayed diagnosis. If successful, initial decompression of the stomach through a nasogastric or orogastric tube will abort the emergency situation, to be followed by a definitive repair of the diaphragmatic defect.

## References

[1] Thal ER , Friese RS . Traumatic Rupture of the Diaphragm. In: Fischer JE, Bland KI, eds. Mastery of Surgery. 5th edn. Philadelphia: Lippincott Williams & Wilkins; 2006 pp634–641

[2] McCann B , O’Gara A . Tension viscerothorax: an important differential for tension pneumothorax. Emerg Med J 2005; 22: 220–221 15735278 10.1136/emj.2003.008367 PMC1726701

[3] Mizobuchi T , Iwai N , Kohno H *et al.* Delayed diagnosis of traumatic diaphragmatic rupture. Gen Thorac Cardiovasc Surg 2009; 57: 430–432 19779793 10.1007/s11748-009-0418-0

[4] Yates AM , Fulcher JW , Ward ME . Sudden death following delayed traumatic diaphragmatic herniation. Am J Forensic Med Pathol 2011; 32: 47–49 20683321 10.1097/PAF.0b013e3181ed7a13

[5] Khan MA , Verma GR . Traumatic diaphragmatic hernia presenting as a tension fecopneumothorax. Hernia 2011; 15: 97–99 20054598 10.1007/s10029-009-0620-0

[6] Kamiyoshihara M , Ibe T , Takeyoshi I . Chilaiditi’s sign mimicking a traumatic diaphragmatic hernia. Ann Thorac Surg 2009; 87: 959–961 19231439 10.1016/j.athoracsur.2008.07.033

[7] Bagheri R , Tavassoli A , Sadrizadeh A *et al.* The role of thoracoscopy for the diagnosis of hidden diaphragmatic injuries in penetrating thoracoabdominal trauma. Interact Cardiovasc Thorac Surg 2009; 9: 195–197 19470502 10.1510/icvts.2008.195685

[8] Shiraki K , Hamada M , Sugimoto K *et al.* Pneumothorax after diagnostic laparoscopy. Hepatogastroenterology 2002; 49: 1,033–1,035 12143195

[9] Ahn S , Kim W , Sohn CH , Seo DW . Tension viscerothorax after blunt abdominal trauma: a case report and review of the literature. J Emerg Med 2012 1 12 [Epub ahead of print.] 10.1016/j.jemermed.2011.05.084 22244294

[10] Onakpoya U , Ogunrombi A , Adenekan A , Akerele W . Strangulated tension viscerothorax with gangrene of the stomach in missed traumatic diaphragmatic rupture. ISRN Surg 2011; 458390 22084758 10.5402/2011/458390 PMC3199939

[11] Nishijima D , Zehbtachi S , Austin RB . Acute posttraumatic tension gastrothorax mimicking acute tension pneumothorax. Am J Emerg Med 2007; 25: 734.e5–734.e6 17606109 10.1016/j.ajem.2006.12.019

[12] Slater RG . Tension gastrothorax complicating acute traumatic diaphragmatic rupture. J Emerg Med 1992; 10: 25–30 1629587 10.1016/0736-4679(92)90006-f

